# Ruminant Production from Napier Grass (*Pennisetum purpureum* Schum): A Review

**DOI:** 10.3390/ani14030467

**Published:** 2024-01-31

**Authors:** Mohammed Rafiqul Islam, Sergio C. Garcia, Md. Ashraful Islam, Md. Khairul Bashar, Anamika Roy, Biplob Kumer Roy, Nathu Ram Sarker, Cameron Edward Fisher Clark

**Affiliations:** 1 Livestock Production and Welfare Group, Faculty of Science, The University of Sydney, Camden, NSW 2570, Australia; md.islam@sydney.edu.au (M.R.I.); sergio.garcia@sydney.edu.au (S.C.G.); 2 Dairy Science Group, Faculty of Science, The University of Sydney, Camden, NSW 2570, Australia; 3 Department of Dairy Science, Faculty of Animal Science and Veterinary Medicine, Patuakhali Science and Technology University, Babuganj, Barishal 8210, Bangladesh; sohel238@pstu.ac.bd; 4 Bangladesh Livestock Research Institute, Savar, Dhaka 1341, Bangladesh; kbashar20@yahoo.com (M.K.B.); biplobkumerroy@gmail.com (B.K.R.); 5 Department of Livestock Services, Krishi Khamar Sarak, Farmgate, Dhaka 1215, Bangladesh; royanamikadls30@gmail.com; 6 Krishi Gobeshona Foundation, Bangladesh Agricultural Research Council Complex, Farmgate, Dhaka 1215, Bangladesh; sarkernr62@yahoo.com

**Keywords:** food security, best management practice, elephant grass

## Abstract

**Simple Summary:**

Napier grass is a feed commonly used across the tropics and subtropics for dairy and meat production. Despite this widespread Napier grass use, the current quality of this feed is low alongside the resultant levels of production. However, when Napier grass is managed well, moderate levels of milk and meat can be produced without grain-based supplementation. Such Napier grass management across the tropics and subtropics would transform production systems, markedly increasing animal-sourced protein production.

**Abstract:**

Napier grass (*Pennisetum purpureum* Schumach) supports a significant proportion of animal production in subtropical and tropical regions, but its quality is low and when offered alone, results in low ruminant production. Shifting the management of Napier grass towards a higher-quality feed increased milk yield and liveweight gain for small, mature cattle without supplementation. This review highlights the opportunity for further increases in milk and meat production for differing classes of livestock in the tropics and subtropics by improving the nutritive value of Napier grass using new best management practice flowing on to improve food security for the millions of people in these regions.

## 1. Introduction

Many countries in the tropics and subtropics are seeking to increase livestock productivity in line with increasing income, population and the associated demand for animal-sourced protein [1,2]. This greater consumption of animal protein has, in part, been supported by ruminants offered tropical feeds such as Napier grass. For instance, Bangladesh’s milk and meat production increased from 5.1 to 13.1 million tons (17.6% increase/yr) and 3.6 to 9.3 million tons (17.3% increase/yr), respectively, from 2013 to 2022, while the number of cattle was maintained (23.3 to 23.9 million) during the same period [3]. The shift toward crossbreeds from indigenous cattle was one of the main drivers for this increased production, with approximately 30% of the total cattle now being crossbred [4]. In a survey in Southwestern Bangladesh consisting of 250 farmers, it was reported [5] that the herd size of cattle per farm was 6.8, of which 5.9 and 0.9 cattle were crossbred and indigenous cattle, respectively. The average milk production of these cows was 8 and 2 litres (L)/cow/day (farm average 6.5 L/cow/day), respectively. Another survey consisting of 70 farms (7–18 cows/farm; average 10) in Central Bangladesh reported [6] 100% crossbred cows in that area, with a milk yield across farms of 10 L/cow/day ranging from 6 to 14 L/cow/day. Similarly, in a survey of 30 beef-fattening farmers in an area in Central Bangladesh [7], 88% of the total cattle were crossbred, and 83% of these farmers use cultivated grasses. This scenario is like many other tropical and subtropical countries where farmers are shifting towards rearing non-indigenous animals [8].

Napier grass (*Pennisetum purpureum* Schumach) comprises up to 80% of the forage ingested by cows in many tropical and subtropical countries [9], but this grass is low in nutritive value under the current management and often cannot support animal productive performance [10,11]. As such, Napier grass management for feed quality improvement could markedly improve the level of animal production. A recent review [12] reported that the crude protein (CP) content of this grass can be increased from 96 to 257 g/kg dry matter (DM), and the metabolisable energy (ME) content can be increased from 8.7 to 10.8 mega joules (MJ)/kg DM by simple changes in defoliation height. This suggests that high-quality Napier grass containing CP 200 g/kg DM and ME 10 MJ/kg DM is possible in line with the requirements for moderate levels of milk and meat production per animal in the tropics and subtropics and high (>25,000 L milk/ha) levels of production per hectare [13]. Therefore, Napier grass has the potential to aid in achieving food security and transform the lives of millions of people in most tropical and subtropical countries around the world [12,14]. As such, research and adoption programmes on improved management of Napier grass are urgently required to support the World Bank’s programme vision to end malnutrition and associated stunting and wasting by 2025–2030 in tropical and subtropical regions [14].

This review will analyse current animal production using Napier grass, identify gaps and provide direction on how to manage and offer this grass to substantially increase the production of cattle across the tropics and subtropics where this grass is prevalent.

## 2. Data

The literature was searched from CAB Abstract via Web of Science (1973–present; https://libguides.library.usyd.edu.au/az.php?a=c (accessed on 12 January 2023)). The searched topic was ‘Napier grass’, filtered by Publication Years ‘1980–2023’, Document Types ‘Journal Article’, CABICODES ‘Animal Nutrition Production Responses’ and ‘Dairy Animals’. This search generated 303 journal articles, of which 75 were open access and 62 CABI Full Text. Out of these, only 20 articles were related to intake and/or digestibility and production performances, where Napier grass was offered ad libitum for ruminant production, which were open access and included in this review.

## 3. Current Animal Production Systems from Napier Grass

### 3.1. Dairy Cows

Feed intake is a key determinant of dairy cow milk production. Napier grass usually contains a high percentage of neutral detergent fibre (NDF), which limits ruminant DM intake. On average, low-producing dairy cows can consume DM and NDF at 2.1% and 1.8% of their liveweight (LW), respectively, and 42.9 MJ ME/day from Napier grass containing 84 g CP/kg DM, 723 g NDF/kg DM and 7.5 MJ ME/kg DM (Table 1). Napier grass of this quality (i.e., low CP and ME, and high NDF) and intake supported the production of 7.9 kg milk/day/cow (but continued to lose its LW at 36 g/day), a reduction in the average 10.2 kg milk/day before the start of the experiment (Table 1). This suggests that in addition to quality, DM intake from this grass should be increased beyond 2.1% to sustain milk production of cows either by reducing the NDF content of this grass or by supplementing feed which has less NDF content. The total DM intake is limited when NDF intake is greater than 1.2% of LW [15]. However, it is difficult to manage NDF intake at this level in tropical C_4_ grass-based diets, as NDF content from these grasses is generally high, and consequently, NDF intake on grass-based diets is also likely to be high. Cows consume NDF, on average, at 1.6% of LW when the total intake is 20.4 kg DM/day (17.4 kg from forages and 3 kg from grains; DM intake 3.3% of LW) from kikuyu grass (*Pennisetum clandestinum*) containing 203 g CP/kg DM and 470 g NDF/kg DM [13]. A similar NDF intake (1.6% on average, 1.2–1.9% of LW) was calculated from elsewhere [16]. Therefore, feed quality, particularly CP and ME, should be increased, and NDF should be decreased in Napier grass to increase DM intake by cows beyond 2% of LW to sustain milk or meat yield.

The Napier grass used for milk production had a harvest interval (HI) of between 70 and 91 days and contained 60–80 g CP/kg DM and approximately 7.5 MJ ME/kg DM [17]. As a consequence, the cows failed to sustain their LW and milk production. Napier grass harvested between 70 and 91 days HI contained 61–72 g CP/kg DM, 714–786 g NDF/kg DM, and 455–503 g ADF/kg DM (7.76 MJ ME/kg DM calculated), and 576 g/kg DMD was offered ad libitum (with ad libitum mineral block lick) to Friesian cows (370–405 kg LW; yielding 15 kg milk/cow/day; body condition score, BCS 3) for 37 days [17]. Dry matter intake from this grass was 8.4–9.1 kg DM/cow/day (2.3% of LW), and NDF intake was 1.75% of LW. However, cows offered feed of this quality were not able to sustain milk production and LW, resulting in a production of 6.8–7.9 kg 4% fat-corrected milk/cow/day (compared to 15 kg/cow/day at the start of the experiment) alongside a loss of 530–890 g LW/cow/day by the end of the experiment [17]. Maintaining 15 kg milk and 220 g LW gain per day would have required 1085–1227 g CP/cow/day and 138–145 MJ ME/cow/day, but the cows received 512–655 g CP/cow/day and 71–75 MJ ME/cow/day from Napier grass harvested at 70–91 days HI [17]. Similarly, Friesian cows (424–441 kg, 4 years old, 210 days in milk, 15 kg milk/cow/day) needed 8 kg concentrate/cow/day (containing CP 160 g/kg DM, ADF 100 g/kg DM, and ash 50 g/kg DM) to maintain 15 kg milk/cow/day when Napier grass was offered after harvesting at 55 days HI containing 86 g CP/kg DM and 410 g ADF/kg DM [21]. In the same experiment, milk yield dropped to 10.5 kg/cow/day from 15 kg within 4 weeks of the experiment when this grass was offered ad libitum with minerals at 0.05 kg/100 kg LW. These cows needed another 4 weeks to reach a normal state of milk production (i.e., 15 kg milk/cow/day) when 8 kg concentrate/cow/day was offered with Napier grass [21]. Thus, farmers currently supplement a large amount of imported (to the farm), higher-density feed protein and energy when Napier grass aged 70–91 days is offered to cows to maintain 15 kg milk/cow/day.

In another experiment conducted over 14 weeks using Ayreshire/Brown Swiss × Shahiwal cows (430 kg LW, 10 kg/cow/day, 1–4 lactation stage, 14 days in milk), Napier grass harvested either at 1 m (28–42 days HI) or 1.5 m (>42 days HI) plant height was offered to cows supplemented with mineral blocks ad libitum [10]. The crude protein content of these grasses was 72 and 56 g/kg DM, and ME (MJ/kg DM, calculated) was 7.6 and 7.0, respectively. The 1 m Napier grass treatment had a greater intake (9.1 vs. 6.5 kg DM/cow/day) and milk yield (8.5 vs. 6.1 kg/cow/day) compared to the 1.5 m plants, and the cows in the 1.5 m Napier grass treatment could support 60% of the total milk (i.e., 6.1 kg) compared to their yield before the start of the experiment (i.e., 10 kg) [10]. In the same work, milk yield decreased by 330 and 220 g/week in cows fed Napier grass harvested at 1.5 m and 1 m, respectively. In addition, cows in both groups lost weight (−420 vs. −665 g/cow/day) [10]. Supplementation of *Leucaena leucocephala* even up to 8 kg/cow/day (fresh) was not able to increase the milk yield or LW of the cows, although weight loss was reduced to −20 g/cow/day in the 1 m height group compared to −325 g/cow/day in the 1.5 m height group [10]. Similarly, Jersey cows offered Napier grass (1.5 m plant height, CP 64–67 g/kg DM, 500–530 g/kg digestible organic matter in DM, DOMD) ad libitum over 50 days were able to produce 4.2–6.4 kg milk/cow/day, and the cows failed to maintain LW (losing 4–29 g/cow/day) [20]. Supplementation of 300 g CP, either by fish meal (980 g/cow/day) or copra meal (1220 g/cow/day), increased milk yield by 1.2–1.6 kg/cow/day, but the cows were still losing 23–25 g LW/cow/day [20]. Thus, Napier grass offered to cows when harvested at 28 days or greater HI, and 1 m or greater height, was not able to supply the protein and energy required for moderate levels of milk production.

In contrast, an experiment with kikuyu grass, a C_4_ grass and a *Pennisetum* species that belongs to Napier grass, reported high DM intake (12–18 kg/cow/day) and milk yield (16–27 L/cow/day), despite the high NDF intake (1.6–2.2% of LW) [22]. Such high DM and NDF intakes were achievable due to high DM digestibility (DMD, 640–780 g/kg), particularly acid detergent fibre (ADF) digestibility (ADFD, 484–688 g/kg DM), as grasses were grazed at an early growth stage [23]. Cows can eat up to 1.6% NDF of LW when fibre digestibility is high [24]. However, the intake of excess fibre by ruminants is undesirable as it is associated with energy costs in the digestive process, the consequences of which include greater ME requirements for maintenance [25,26]. The digestibility of NDF is also important as each unit increase in forage NDF digestibility can increase the DM intake of cows by 0.17 kg, which can increase milk yield by 0.23 kg [27]. These data indicate that grasses offered at an early stage of growth increase fibre digestibility which increases the intake, digestibility and productivity of animals. 

In line with the experience of kikuyu grass, Napier grass grazed (or harvested) by cows at 30 days HI does not need any supplement for most of the year, except in dry seasons for moderate milk production in a smallholder production system. In an experiment [18] conducted over 1 year, Holstein–Zebu cross cows (483 kg LW, 45–60 days in milk) grazed solely Napier grass at 30 days HI, and at a 4.5 cows/ha stocking rate, produced on average 11.4 kg milk/cow/day. The intake of the cows ranged from 12.9 to 14.9 kg DM/cow/day (2.7–3.0% of LW; calculated NDF intake of 1.8–2.0% of LW), and the consumed grass contained 112–137 g/kg DM CP, 690–750 g/kg DM NDF, 380–440 g/kg DM ADF and 530–580 g/kg IVDMD (in vitro DM digestibility) [18]. This result is supported by other works [28,29], who reported mid-lactating cows can produce 14–15 L milk/cow/day when well-managed kikuyu grass supplemented with minerals is offered, provided DM intake (13 kg DM/cow/day) is not limited by its NDF content (when NDF intake does not exceed 1.5% of LW). Therefore, more research is required to investigate if cows can produce >10 kg milk/day and maintain LW when Napier grass is managed to contain >14% CP.

### 3.2. Dairy Goats

Similar to cows, when a sole diet of Napier grass is offered to dairy goats, milk production typically declines. Napier grass harvested at 49 days HI containing 113 g CP/kg DM, 696 g NDF/kg DM, 600 g/kg DMD and 10.2 MJ ME/kg DM was offered to Toggenburg goats (2 years old, 29 kg LW, 9 months of lactation, 3rd month of pregnancy, 430 g milk/goat/day) over 11 days [11]. The intake of this grass was 1.21 kg DM/day (4.2% of LW). However, the goats were able to maintain 117 g milk/goat/day (out of 430 g milk/goat/day) and a complete drying up occurred during the end of the trial. The calculated protein and digestible energy requirements per goat were 191 g and 20 MJ per day, but each goat was receiving 163 g and 13 MJ per day, respectively [11]. Thus, Napier grass harvested at 49 days HI was not able to supply the CP and ME required by the lactating dairy Toggenburg goats, despite its high intake (4.2% of goats LW). Thus, Napier grass managed under the current management and offered to lactating cows and goats requires a high amount of expensive supplementation (protein, energy and minerals) to produce milk. These include fish meal, molasses and mineral blocks, which smallholder farmers in the tropics and subtropics may not afford to offer. Information is also lacking as to whether such supplementation is cost effective. Therefore, current Napier grass management for the milk production of cows, goats or other lactating animals needs to be altered towards an improved management strategy to increase protein and energy to limit the requirement for expensive supplements. 

### 3.3. Cattle and Buffalo Growth

Napier grass (56 days HI, 12 t DM/ha/year grown with 110 kg N/ha/year) containing 117 g CP/kg DM, 560 g NDF/kg DM and 704 g/kg DM potential degradability (PD, 96 h; 550 g/kg DM effective degradability, ED) was offered to Friesian or Shahiwal heifers (163–181 kg LW; 1 year old) over 120 days [30]. Heifers grew at 390 and 420 g/day, respectively, solely on Napier grass. The DM intake was 5.6–6.0 kg/cow/day (3.4% of LW), and the estimated NDF intake was 1.8–1.9% of LW for both groups [30], similar to the intake of cows per unit of LW reported in the literature (NDF intake 1.6–2.0% of LW) [17,18]. Despite the high intake, this growth (i.e., 390–420 g/day) [30] was low, since the ARC standard [31] indicates that grasses containing 120 g CP/kg DM would allow heifers to gain 500 g/day as they would meet the rumen degradable protein requirement of heifers for this growth. Nonetheless, this growth (i.e., 390–420 g/day) from the feeding of a sole Napier grass diet at 56 days HI containing 117 g/kg DM CP [30] was greater compared to the 210–250 g/day growth of young stocks reported by others who offered Napier grass containing <80 g CP/kg DM [32,33]. However, with similar CP (118 g/kg DM) and NDF (587 g/kg DM) content of this grass which was harvested at 0.5 m height (42 days HI; 8.6 MJ ME/kg DM), a 500 g/day LW gain in Holstein heifers (144 kg LW) with intakes of DM and NDF at 3.5% and 2% of LW, respectively, was reported in an experiment conducted over 104 days [34]. This growth of the heifers matches with the growth reported by ARC [31], suggesting cattle solely offered Napier grass containing approximately 12% CP and 55% NDF will grow at 500 g/day.

A greater DM intake (11.6 kg DM; 2.6% of LW) was reported when 70-day-old grass (containing 80 g CP/kg DM, 540 g NDF/kg DM, 8.2 MJ/kg DM) compared to 105-day-old grass (10.1 kg DM, 2.3% of LW; containing 50 g CP/kg DM, 630 g NDF/kg DM, 7.3 MJ/kg DM) was offered to Holstein steers (445 kg LW) [35]. Although the NDF contents of 70- and 105-day-old grasses were 540 and 630 g/kg DM, respectively, the NDF intakes between the grass groups were similar (6.4 kg, 1.44% of LW) [35]. Similarly, a greater DM intake of this grass by buffalo steers (300 kg LW) was reported when 40-day-old grass (8.9 kg DM intake; 3.0% of LW) was offered compared to 60-day-old grass (7.8 kg DM intake; 2.6% of LW) [36]. The NDF contents of those grasses were 721 and 787 g/kg DM, respectively, but NDF intakes were similar between the groups (6.6 kg, 2.2% of LW) [36]. This suggest that animals, cattle or buffalo, young or old, cannot consume NDF beyond a certain threshold, and the main driver to increase DM intake from Napier grass is to reduce its NDF content, as animals are not able to consume sufficient DM due to their rumen fill by NDF from mature (older) grasses. Nonetheless, these intakes of NDF by cattle (1.44% of LW) [35] and buffaloes (2.2% of LW) [36] were greater than the recommended values [15], which reported that the total DM intake is limited when the NDF intake is greater than 1.2% of LW. Also, the NDF intake by buffaloes was greater than that of cattle. Thus, Napier grass containing 12% CP and 8.6 MJ ME/kg DM offered to steers/heifers can enable them to grow from 390 to 500 g/day. Overall, intakes of DM and NDF of 2.4% and 1.4% of steer/heifer LW, respectively, from Napier grass containing 594 NDF/kg DM and 626 g/kg DMD (dry matter digestibility) were able to support 445 g LW gain/day/steer (Table 2).

### 3.4. Goat and Sheep

On average, intakes of DM and NDF of 3.3% and 2.0% of goat/sheep LW, respectively, from Napier grass containing 663 NDF/kg DM and 611 g/kg DMD (dry matter digestibility) were able to support 100 g milk or 54 g LW gain/day (Table 3). However, Napier grass (harvested at 60–70 days HI) containing 83 g CP/kg DM, 673 g NDF/kg DM, 570 g/kg DMD and 7.8 MJ ME/kg DM was offered to Boer × Local goats (12 kg LW) over 82 days [42]. With such quality, these goats were losing 13 g LW/goat/day, although they were consuming DM at 3.4% of their LW (NDF intake 2.2% of LW). However, these goats were gaining 53 g/head/day when Napier grass was supplemented with a concentrate mixture containing 127 g CP/kg DM and 10.5 MJ ME/kg DM. This growth was achieved when the intake of Napier grass was 276 g/day and that of concentrate was 493 g/day (i.e., roughage–concentrate = 36:64; concentrate at 2% of their LW), indicating that a high amount of concentrate is required for growth when Napier grass offered to goats is at high maturity (or HI) [42]. This, taken alongside the work above, suggests that both the protein and energy contents of Napier grass need to be increased, or supplemented, to support the growth of goats, similar to the needs of cattle. Therefore, Napier grass under the current management cannot support the growth of goats without supplementation. Research is needed to offer this grass at a lower maturity and containing >12% CP and >9 MJ ME/kg DM to investigate the growth potential of different species of animals.

## 4. Production System Strategies to Increase Milk and Meat

A dairy cow (600 kg LW) usually requires 170 g CP/kg DM, <450 g NDF/kg DM, 180 g ADF/kg DM, and 10.3 MJ ME/kg DM to sustain 20 L milk/cow/day [28]. Well-managed kikuyu grass offered ad libitum to mid-lactating Holstein cows supplemented with minerals can produce 14 L milk/day based on an intake of 13 kg DM/cow/day of grass, and this intake is not limited by NDF at 1.5% of LW [29]. Kikuyu grass at the 4.5 leaf stage (LS) provides the highest proportion of leaves and the highest quality for cow consumption, after which the proportion of stem increases and the quality decreases [28,46].

An experiment conducted over 2 years adopted this LS principle in a grazing decision and reported 20–24 t DM/ha/year from kikuyu-based grass [13]. Kikuyu-based grass can be grazed (or harvested) at an interval of 10 days in the summer and spring months as it regrows quickly, whereas it can take a month to regrow in winter. A total of 20–24 times grazing per year at a rate of 1 t DM per grazing at 4.5 LS was developed as a thumb rule [13]. Whenever grass grew to 2.5 t DM/ha, 1 t DM/ha was grazed, leaving 1.5 t DM/ha in the field (i.e., stubble) for regrowth to achieve 20–24 t DM/ha grass with the quality required for dairy cows [13]. In an animal experiment conducted over 2 years with 100 Holstein cows (613 kg LW), it was demonstrated that cows were able to produce 27,831 L milk/ha/year from forages only (without any supplements). This milk production was increased to 34,499 L/ha/year with supplements (3 kg grain/cow/day), and the milk yield per cow/lactation (305 days) in this experiment was 7653 L when forage yields were maintained at 26 t DM/ha/year and contained 200 g CP/kg DM and 10.2 MJ ME/kg DM [13]. Therefore, Napier grass yielding 71 t DM/ha containing 135 g CP/kg DM [47] or more and 10.8 MJ ME/kg DM reported elsewhere [47,48,49] has great prospects in tropical and subtropical countries to maximise milk and meat production [12,14]. In line with those evidences, an experiment conducted over 2 years demonstrated that cows fed Napier grass at 30 days HI (containing 140 g CP/kg DM, 690–750 g NDF/kg DM, and 580 g/kg DM digestibility) can produce on average 11 kg milk/cow/day, where the cows maintained an intake of 12.9 kg DM/cow/day (2.7% of LW) [18]. This suggests there is a big opportunity to increase milk production in the tropics and subtropics if Napier grass can be managed to contain >140 g/kg DM CP and close to 10 MJ ME/kg DM. Therefore, harvesting Napier grass at an early growth stage (e.g., 30 days or less HI) could be an option until a practical leaf-based system can be introduced to optimise both yield and nutritive value for further optimisation of milk or meat [12,38].

There is evidence that Napier grass can be managed for a nutritive value close to the nutrient requirements of dairy or beef cattle or other animals for production purposes if managed properly. This grass, at between 14 and 28 days HI, contains CP (190–230 g/kg DM), ADF (280–340 g/kg DM), NDF (490–500 g/kg DM) and ME (11.7 MJ ME/kg DM) [48,49], which is close to the nutritive value required by cows for milk or meat production. The nutritive value of this Napier grass is similar to the nutritive value of kikuyu grass harvested (grazed) at the 4–5 leaf stage [25,28] to achieve 14 L milk/cow/day from mid-lactating cows by grazing this grass without any supplementation [28] or to support up to 30 L of milk with supplementation [22]. Cut and carry at one month intervals may also offer the opportunity to harvest 12 times/year to supply forages year round, compared to the current management of 6–7 harvests/year.

Our calculation/estimation (Table 4) indicates that a 613 kg cow, when its NDF consumption is kept at 1.6% of LW [13], is theoretically likely to be able to consume 18.2 kg DM/day solely from Napier grass, which will be able to supply 211 MJ ME and 4.1 kg CP per cow/day to produce 24 L milk/cow/day based on the composition of Napier grass at a 28-day HI (12 harvests/year) [49]. In contrast, when Napier grass is harvested at an 84-day HI (four harvests/year) [49], although yielding 11 t DM/ha/year, a cow is likely to be able to consume 14.9 kg DM/day (NDF digestibility not considered, which may further lower intake), with a supply of 145 MJ ME and 1.1 kg CP per cow/day (Table 4). Thus, there may be a deficit of 59 MJ ME and 3 kg CP per cow/day to produce 24 L milk/cow/day when grass is harvested at an 84-day HI and offered to a cow (Table 4). Even if farmers wish to supplement, such supplementation to fill the deficit of energy and protein (59 MJ ME and 3 kg CP per cow/day) would be expensive. In addition, as 14.9 kg DM is the limit of intake of grass at an 84-day HI (at NDF intake 1.6% of LW; Table 4), any such supplementation of energy or protein would occur at the expense of the offer or intake of Napier grass, which would further increase the cost of animal production that smallholder farmers in the tropics and subtropics may not afford. Our calculation indicates it is possible to include only 7 kg DM Napier grass in the diet of a cow with such maturity (i.e., an 84-day HI, 77 g CP/kg DM, 640 g NDF/kg DM) [49] (Table 4). The same Napier grass-based diet would require an additional 13 kg of purchased concentrate mixture containing 270 g CP/kg DM and 10.5 ME MJ/kg DM to supply the total protein (4 kg CP/cow/day) and energy (204 MJ ME/cow/day) required to obtain 24 L milk/cow/day from a 613 kg cow (Table 4). Such a diet would also be highly expensive for smallholder producers.

Achieving a concentrate-equivalent Napier grass (i.e., 205 g CP/kg DM, 10.2 MJ ME/kg DM) [48,49] may be able to change such scenarios of milk and meat production in the tropics and subtropics, as 27,835 L milk/ha/year is achievable with 26 t DM/ha/year when forages are managed to contain 200 g CP/kg DM and 10 MJ ME/kg DM [13]. However, more research is required to increase the nutritive value of this grass without compromising the yield to make it attractive to the land-constrained smallholders in the tropics and subtropics. Therefore, studies are required to determine how much yield can be maintained in order to obtain 200 g CP/kg DM and 10 MJ ME/kg DM consistently from this grass. The theory of a compromise between yield and quality needs to be tested for its suitability and profitability on farms as there is a trade-off between these factors. Knowledge on all management factors currently practiced, e.g., fertiliser, water, variety, maturity or HI, and location, should be applied so that the compromise between yield and nutritive quality remains minimal. Therefore, further work on different management strategies is required to obtain both the quality and quantity of this grass required for high milk or meat production in the tropics and subtropics. The evidence in the literature [47] indicates increasing plant density and harvesting frequency (11 times/year) may be an excellent option to increase both yield and quality and to supply this grass year round. These researchers [47] obtained 71 t DM/ha by increasing plant density from the conventional 100 cm × 50 cm to 50 cm × 40 cm, harvested at a 35-day HI, and achieved a relatively high CP (135 g/kg DM) and ME (10.8 MJ/kg DM; IVDMD 755 g/kg). However, there is no information on milk or meat production from this experiment. Thus, it is necessary to conduct experiments to develop management strategies to obtain the maximum yield of Napier grass when the grass is managed to contain >140 g CP/kg DM and >10 MJ ME/kg DM to increase milk and meat production from this grass in the tropics and subtropics.

## 5. Gaps

The following gaps were identified:

(1) Dairy: Napier grass under current management (CP 84 g/kg DM; ME 8.6 MJ/kg DM) typically cannot support moderate levels of milk yield and liveweight of animals, particularly dairy animals. A high amount of supplementation of concentrate containing high protein and energy levels is required when poorly managed grass is offered but still cannot support both the milk production and liveweight of cows. There is no information on the effect of offering this grass containing >140 g CP/kg DM and >8.5 MJ ME/kg DM to dairy animals. It is also likely that the NDF content of mature Napier grass is less digestible compared to younger grass, so this needs to be investigated.

(2) Animal growth/meat: Steers/heifers offered moderate-quality Napier grass containing 120 g CP/kg DM and 8.6 MJ ME/kg DM grow 390–500 g/d. Napier grass under current management cannot support the growth of goats without supplementation. Research is needed offering Napier grass at a shorter harvest interval and containing >120 g/kg DM CP and >9 MJ ME/kg DM to determine the growth potential of different animal species.

## 6. Conclusions

Napier grass is abundant and widely popular amongst smallholder farmers in the tropics and subtropics for its high biomass yield and growth, but its poor feed quality limits the levels of milk and meat production. Harvesting or cut and carrying the grass at an early stage may improve its quality and has the potential to produce 14–24 L of milk/cow/day in Holstein cows and >500 g gain/cattle/day without any supplementation. However, Napier grass currently requires a trade-off between quality and quantity, which could be resolved by investigating systems with increased plant density. Such work is critical to emulate work conducted on kikuyu-based pasture, which has been shown to yield over 22 t DM/ha, CP 200 g/kg DM, and ME 10 MJ/kg DM, and enable >24 L milk/cow/d per year (>7000 L/cow/lactation or >27,000 L/ha). Thus, the development of a best management system for Napier grass is required to optimise both the quantity and quality of milk and meat yields of smallholder farmers in the tropics and subtropics, contributing to the food security of the vast majority of people in these regions.

## Figures and Tables

**Table 1:** The literature ^1^ on Napier grass composition, intake, nutrient utilisation and productive performances by dairy cows.

	Average	Minimum	Maximum	*n*
Chemical composition of offered grass (g/kg DM or as stated)				
Dry matter (DM)	225	170	380	8
Ash	110	93	152	7
Crude protein (CP)	84	41	128	12
Ether extract (EE)	59	59	59	1
Water soluble carbohydrate (WSC)	90	90	90	1
Neutral detergent fibre (NDF)	723	668	786	8
Acid detergent fibre (ADF)	410	256	503	10
Lignin	55	40	70	3
Metabolisable energy (ME, MJ/kg DM)	7.5	7.0	7.9	4
Animal performance				
Liveweight (LW, kg)	406	289	483	12
DM intake (kg/day)	9.1	2.4	12.9	11
DM intake (%LW)	2.1	0.8	3.9	8
NDF intake (kg/day)	1.6	1.6	1.6	1
NDF intake (%LW)	1.8	1.7	1.8	2
CP intake (kg/day)	0.4	0.1	0.6	2
CP intake (%LW)	0.6	0.1	1.1	6
ME intake (MJ/day)	42.9	42.9	42.9	1
Milk yield before trial (kg/day)	10.2	4.2	15.0	4
Milk yield (kg/day)	7.9	6.9	9.3	5
Milk protein (%)	2.9	2.8	2.9	2
Milk fat (%)	3.1	3.0	3.3	2
Liveweight gain or loss, g/day	−36	−890	450	11
Dry matter digestibility (DMD, g/kg)	545	440	576	7
Organic matter digestibility (OMD, g/kg DM)	482	460	504	2
ADF digestibility (ADFD, g/kg DM)	591	525	668	3
NDF digestibility (NDFD, g/kg NDF)	601	545	666	3

^1^ [10,17,18,19,20,21].

**Table 2:** The literature ^1,2^ on Napier grass composition, intake, nutrient utilisation and liveweight gain by cattle and buffalo.

	Average	Minimum	Maximum	*n*
Cattle				
Chemical composition of offered grass (g/kg DM or as stated)				
Dry matter (DM)	178	149	239	7
Ash	159	112	216	13
Crude protein (CP)	101	39	173	12
Ether extract (EE)	34	27	38	3
Neutral detergent fibre (NDF)	594	540	648	12
Acid detergent fibre (ADF)	325	290	360	9
Lignin	40	26	52	10
Silica	53	53	53	1
Total oxalate	9	9	9	1
Calcium (Ca)	2.3	0.4	3.4	3
Phosphorous (P)	2.1	0.2	3.4	3
Metabolisable energy (ME, MJ/kg DM)	7.9	7.1	8.6	5
Animal performance				
Liveweight (kg)	253.0	119	445.1	9
DM intake (kg/day)	6.1	2.8	11.6	7
DM intake (%LW)	2.4	1.7	3.4	12
NDF intake (kg/day)	2.4	1.6	3.3	4
NDF intake (%LW)	1.4	1.0	2.0	11
Liveweight gain, g/day	445	390	500	2
Dry matter digestibility (DMD, g/kg)	626	496	701	6
Organic matter digestibility (OMD, g/kg DM)	546	525	571	3
Buffalo				
Chemical composition of offered grass (g/kg DM or as stated)				
Ash	67	49	99	4
Crude protein (CP)	98	71	127	4
Ether extract (EE)	754	706	791	4
Neutral detergent fibre (NDF)	458	408	499	4
Lignin	99	74	129	4
Animal performance				
Liveweight (kg)	300	300	300	1
DM intake (kg/day)	7.7	6.5	8.9	4
DM intake (%LW)	2.6	2.2	3.0	4
NDF intake (kg/day)	5.8	5.1	6.6	4
NDF intake (%BW)	1.9	1.7	2.2	4
DMD (g/kg)	538	489	615	4
NDF digestibility (NDFD, g/kg NDF)	556	511	598	4
NDFD (in vitro/in situ, g/kg NDF)	607	527	658	4
In vitro DMD (g/kg)	647	587	681	4

^1^ cattle [30,34,35,37,38,39,40,41]; ^2^ buffalo [36].

**Table 3:** The literature ^1,2^ on Napier grass composition, intake, nutrient utilisation and productive performances by goats and sheep.

	Average	Minimum	Maximum	*n*
Chemical composition of offered grass (g/kg DM or as stated)				
Dry matter (DM)	180	166	194	2
Ash	118	82	137	6
Crude protein (CP)	113	71	136	8
Ether extract (EE)	53	53	53	1
Neutral detergent fibre (NDF)	663	610	708	7
Acid detergent fibre (ADF)	387	372	412	5
Lignin	34	28	49	5
Soluble oxalate	14.7	14.7	14.7	1
Calcium (Ca)	4.3	4.3	4.3	2
Phosphorous (P)	4.4	4.4	4.4	1
Magnesium (Mg)	47	47	47	1
Metabolisable energy (ME, MJ/kg DM)	8.5	7.4	10.2	3
Animal performance				
Liveweight (kg)	21	11	29	3
DM intake kg/day	0.67	0.39	1.21	3
DM intake %BW	3.3	2.9	4.2	6
NDF intake kg/day	0.3	0.3	0.3	1
NDF intake %BW	2.0	1.8	2.2	5
Milk yield before trial, kg/day	0.4	0.4	0.4	1
Milk yield, kg/day	0.1	0.1	0.1	1
Liveweight gain, g/day	54.2	−12.7	121.0	2
DMD in situ (g/kg)	537	537	537	1
DMD (g/kg)	611	570	650	7
OMD (g/kg DM)	659	645	673	2
CP digestibility (g/kg DM)	625	527	714	3
NDF digestibility (NDFD, g/kg NDF)	672	578	740	5

^1^ goat [11,42,43]; ^2^ sheep [44,45].

**Table 4:** Potential impact of harvest interval (HI, 28 or 84; [49] Sileshi et al., 1996) of Napier grass on protein and energy supplement required by dairy cows (based on dry matter and NDF intake [13] Farina et al., 2011).

Parameters ^1^	Sileshi et al. (1996) [49]	Sileshi et al. (1996) [49]	Farina et al. (2011) [13]
Harvest interval (HI, days)	28	84	15–18
Yield (t DM/ha)	5.3	11.1	26.0
CP yield (kg DM/ha)	1174	858	5278
ME yield (kg DM/ha)	61,093	108,327	260,000
CP (g/kg DM)	223	77	203
ADF (g/kg DM)	290	440	
NDF (g/kg DM	527	640	470
WSC (g/kg DM)			9.5
IVDMD (g/kg)	803	690	
ME (MJ/kg DM)	11.6	9.7	10.0
DM intake (kg/cow/day)	18.2	14.9	20.4
NDF intake (kg/cow/day)	9.6	9.5	9.6
NDF intake (%BW)	1.6	1.6	1.6
CP intake (kg/cow/day)	4.1	1.1	4.1
ME intake (MJ/cow/day)	211	145	204
CP supplement required (kg/cow/day)	0.1	3.0	0.0
ME supplement required (MJ/cow/day)	−7.1	59.0	0.0

^1^ DM, dry matter; CP, crude protein; ME, metabolisable energy; ADF, acid detergent fibre; NDF, neutral detergent fibre; WSC, water soluble carbohydrate; IVDMD, in vitro DM digestibility.

## Data Availability

The data supporting the conclusions of this article will be made available by the authors, without undue reservation.
